# An Evidence-based Approach toward a Sustainable Healthcare System

**DOI:** 10.31662/jmaj.2018-0014

**Published:** 2019-02-20

**Authors:** Rintaro Mori

**Affiliations:** 1 Department of Health Policy, National Center for Child Health and Development, Tokyo, Japan

**Keywords:** sustainability, health system, evidence-based health policy, population aging

## Abstract

Japan is the most rapidly aging country in the world, and the sustainability of its health and social care system is a top priority. In order to have a sustainable healthcare system, global protection of healthcare commons through regulations, together with a market mechanism based on societal values, is critical. An evidence-based approach is needed to attain that; however, the current methodologies for this approach have major limitations, such as the lack of common healthcare goals, the retrospective nature of evidence, and the uncertainty and ambiguity of the available data. This opinion paper discusses the challenges in developing a sustainable system and proposes a feasible way to overcome the limitations.

## Sustainable Healthcare System

While the aging population phenomenon is becoming more common around the world, Japan has the highest proportion of elderly people, with 27.3% of its population being 65 years of age or older in 2017 ^(1)^. A significant implication of an aging population is the financial burden of social security and healthcare. To that end, Japan needs to critically revise its healthcare system to ensure the sustainability of its population.

Sustainable development is defined as a “development that meets the needs of the present without compromising the ability of future generations to meet their own needs” in the 1987 Brundtland Commission Report ^(2)^. Although the report popularized this definition, a notable criticism of the report was that it presented the desire to realize a sustainable system without actually describing a realistic path to achieve it.

One of the major challenges in establishing a sustainable system is achieving a primary balance, regardless of whether it employs a tax- or insurance-based system. Furthermore, this challenge is compounded by the difficulty of introducing new innovations to the market and the inflexibility of medical professionals to adopt such new approaches.

In order to facilitate the process of introducing effective (including cost-effective) interventions and eliminating ineffective (non-cost-effective) interventions in the market, health technology assessments and/or clinical practice guidelines (HTA/CPG) have been widely adopted in some countries ^(3)^. Health technology assessment “refers to the systematic evaluation of properties, effects, and/or impacts of health technology” from the social, economic, organizational, and ethical perspectives. Clinical practice guidelines include a wider scope with a similar methodology, providing more comprehensive guidance on a particular health condition. While clinical practice addresses the individual level, all HTAs/CPGs should be based on population studies.

To ensure a sustainable system, it is critical to protect the “healthcare commons,” which are the common areas related to health that are owned and regulated by the public following the argument around the “Tragedy of the Commons ^(4)^.” When the commons are abused beyond a certain level, the system loses its functions. In order to protect such commons, effective regulations are necessary ^(5)^. Here, we look at what constitutes a sustainable healthcare system and how it can be achieved.

## Methods to Develop/Assess Cost-effective Policies and Regulations to Protect Commons

Evidence-based medicine utilizes the best evidence from well-designed research to assist in decision-making in patient care. This had a significant impact on healthcare in every aspect, including global health ^(6)^.

## Development of Evidence-based Approaches to Global Health

The theoretical basis of evidence-based medicine was developed from the premise that clinical practice should not rely on the “craftsman judgments” of health professionals, but rather on rational decision-making based on the best available scientific data. In other words, decisions made by health professionals should be, as much as possible, based on evidence from high-quality research. A 2007 survey of over 1,000 systemic reviews revealed that 44% concluded that interventions studied were likely to benefit clinical practice ^(7)^. In spite of this, a huge gap persisted between scientific research and clinical practice, and the results of many studies were not applied in actual practice. Instead of receiving optimal treatments based on well-collected and validated evidence, patients may receive “second-best” therapies based on craftsman judgments, categorized as “overuse” of healthcare interventions, “underuse” of other interventions, and “misuse” of interventions ^(8)^.

Nonetheless, evidence-based medicine has emerged as the main tool for health policy decisions since the 1990s. Governments worldwide have been devising health research strategies, coordinating research activities, and allocating funding in a more consistent way to serve specific research and development goals. Research syntheses, meta-analyses, and systematic reviews have become the main methods for gathering validated evidence in global health policy research. One of the most notable milestones in the evidence-based health policy movement was the establishment of the UK Cochrane Centre in 1992, which fortifies the notion that evidence-based medicine is central to making health policy decisions that are rational and cost-effective.

Consequently, policymakers in the field, including bilateral and multilateral donors, development banks, United Nations agencies, national governments, and nongovernmental organizations, have stepped up their efforts to ameliorate health outcomes in targeted communities by exploiting the best available evidence. Indeed, many countries using evidence-based approaches in health policy have witnessed positive transformations. For example, Mexico underwent a major reform in its healthcare system using evidence from systematic analyses and the experiences of other countries, together with other measurement methods ^(9)^.

Currently, there are two pillars of evidence-based approaches to global health. The first is to develop health policies based on syntheses of comparative effectiveness research, namely, randomized trials, systematic reviews, and cost-effectiveness analyses. Cochrane is the prime example of this. The other approach is policy evaluation based on robust epidemiological data, including vital statistics and large surveys, with the Global Burden of Disease project being a prominent example.

## The Future of Global Health and Limitations of Evidence-based Approaches

Fundamental changes of societal, demographical, and environmental structure have occurred on a global scale since evidence-based medicine gained grounds. Ongoing shifts in ecology, economy, demography, and epidemiology all present serious threats to global health, including global warming, development and inequality, aging populations, and chronic conditions such as obesity and diabetes. Given the rapid pace of these changes, traditional global health tools to fight emerging diseases are no longer competent and sustainable.

### Limitation 1: difficulty in establishing a common goal

It is critical to consider the objects and metrics of global health to address imminent problems. In particular, in the face of economic uncertainties, policymakers have to focus on improving efficiency. If the goal for a better system somehow differs among key players, even ever so slightly, the pursuit of a common goal could go astray. Therefore, a more realistic goal for global health is needed, one that requires an intergenerational approach with measures of population well-being that transcend common metrics such as Disability-Adjusted Life Years (DALYs) or Quality-Adjusted Life Years (QALYs).

Although DALYs and QALYs have been employed in the Global Burden of Disease project, they are not necessarily aligned with global health goals ^(10)^. These metrics may contain useful information, but they should be used with caution. Such a discussion can be made on the basis of the WHO (World Health Organization) ’s definition of health, which is described as “a state of complete physical, mental, and social well-being and not merely the absence of disease or infirmity ^(11)^.” The simplicity and rigorousness of DALYs and QALYs lead to an inherent lack of diversity in the conception of well-being or happiness, including those of future generations, which are hard to quantify, since the measures narrowly focus on what is simple to calculate, particularly regarding sustainable developmental goals ^(12)^. One example is in palliative care, particularly in the context of end-of-life care. These metrics may contain useful information, but their use has been so dominant that they have supplanted the more meaningful goal that they were designed to achieve. Such a discussion can be made on the basis of the WHO’s definition of health, which is described as “a state of complete physical, mental and social well-being and not merely the absence of disease or infirmity ^(11)^.” Hence, discussions should abound if the best interest of patients will not necessarily cause an increase in QALYs and DALYs. Another example is in maternal and child health policy interventions, in which these indicators are often not considered in strategies to improve health and welfare of ensuing generations.

### Limitation 2: retrospective nature

A fundamental limitation is the “backward-looking” disposition of evidence-based approaches, as the best available data rely on historical research. The rapid development of therapeutic procedures may render the information collected through an evidence-based approach obsolete. Furthermore, evidence-based approaches do not evaluate emerging or future trends, although the immediate ensuing decades should be the main period of time that global health policy is expected to influence. The current emphasis and debate on chronic conditions and mental health of middle-aged populations particularly highlight the notion that such an approach cannot promote the health of future generations and that it is ill-equipped to gage the importance of supporting the reproductive cycle of people. With that in mind, and in addressing the sustainability of the reproductive cycle of populations in developing countries, current evidence-based health policies may even contradict its goals.

### Limitation 3: data ambiguity

Another major limitation of the evidence-based approach is its uncertainty in its data, particularly its increasing reliance on randomized trials. Evidence-based approaches may not be able to answer important questions such as “What does dignity mean for humankind?” While these limitations are recognized by academics, many policymakers underestimate the effects of these problems, although these are external limitations.

## The Way Forward

Considering these limitations and their implications, we should develop a new metric that would capture the comprehensive concept of health and well-being, which can be applied to all sectors. This can be achieved by modifying such indicators to include common features that appeared in subjective well-being and/or by setting up an interpretation framework to interpret the results of QALYs and DALYs, considering their limitations as a bridge to the recommendations. As for the ambiguity and retrospective nature of the available data, consensus development measures, including stakeholder involvement with effective management of conflict in both financial and nonfinancial interests, can control the problems within data limitations to a certain degree. Overall, a more comprehensive and holistic approach to evidence-based measures is warranted ^(5)^.

## Article Information

### Conflicts of Interest

None

### Acknowledgement

I sincerely appreciate Drs. Kayo Onishi, Julian Tang, and Jesse Bump for their intellectual contribution to the discussion and manuscript.
